# The role of vital signs in predicting mortality risk in elderly patients visiting the emergency department

**DOI:** 10.1186/s12873-025-01307-8

**Published:** 2025-08-01

**Authors:** Karin Erwander, Björn Agvall, Kjell Ivarsson

**Affiliations:** 1 Department of Clinical Sciences, Lund University, Lund, Sweden; 2 Department of Research and Development, Region Halland, Halmstad, Sweden; 3 Center for Primary Health Care Research, Department of Clinical Sciences, Malmö, Sweden

**Keywords:** Older adults, Emergency department, Geriatric emergency medicine, Vital signs

## Abstract

**Background:**

Accurate risk stratification in older adults presenting to the emergency department (ED) is essential but challenging due to atypical presentations and age-related physiological changes. While vital signs are central to triage, their predictive value for short-term mortality in this population remains unclear. This study aimed to explore the association between initial vital signs and 7-day mortality among older ED patients.

**Method:**

This retrospective cohort study included patients aged ≥ 65 years who visited two EDs in Region Halland, Sweden, during 2018. Vital signs, systolic blood pressure (SBP), heart rate (HR), peripheral oxygen saturation (SpO₂), respiratory rate (RR), body temperature, and level of consciousness (LOC), were extracted from the regional health information system. Descriptive statistics, ROC curve analysis, and logistic regression were used to assess associations between categorized vital signs and 7-day mortality, adjusting for age, sex, and comorbidity.

**Results:**

Of 30 327 ED visits, 25 450 patients had at least one recorded vital sign. The mean age was 78 years; 50% were female. The 7-day mortality rate was 2%. ROC analysis showed limited discriminative ability of individual vital signs (AUC range: 0.49–0.70). Low SBP, high HR, low SpO₂, and impaired LOC were statistically significantly associated with mortality. Multivariable logistic regression confirmed these associations, with SBP (≤ 80 mmHg, AOR 7.79; 95% CI 3.27–18.54), high HR (> 125 bpm, AOR 6.37; 95% CI 1.26–32.16), low SpO₂ (≤ 80%, AOR 3.64; 95% CI 1.75–7.58), and impaired LOC (GCS < 13 (AOR 9.30-18.36)) showing the strongest effects.

**Conclusion:**

Low SBP, elevated HR, reduced SpO₂, and impaired LOC were independently associated with short-term mortality in older ED patients, though their individual predictive performance was modest. LOC, despite being a strong predictor, was frequently missing, underscoring challenges in routine assessment. These findings highlight the need for more comprehensive, geriatric-informed triage systems that incorporate age-related physiological changes and ensure consistent evaluation of LOC. As frailty and functional status was not available in this dataset, future studies should focus on integrated models that include these factors to improve early risk identification in emergency care for older adults.

**Clinical trial number:**

Not applicable.

## Background

The management of older adults at the Emergency Department (ED) have become increasingly important as the population ages and number of ED visits made by elderly patients rise [1, 2]. Older adults in the emergency department present a significant challenge for healthcare providers, as comorbidities, atypical symptom presentation, frailty, and age-related physiological changes can obscure clinical signs and complicate accurate triage. These factors make it more difficult to assess severity, prioritize care, and predict clinical outcomes effectively [1, 3, 4]. Older patients are at risk for adverse events at the ED due to undertriage and lack of specific protocols [1, 5]. Many triage systems commonly used in ED and prehospital settings, such as the Rapid Emergency Triage and Treatment System (RETTS), the Manchester Triage System (MTS), and the Emergency Severity Index (ESI), were primarily developed for younger or general adult populations. Consequently, they may not fully capture the complexities associated with aging, including physiological changes, atypical symptom presentations, and frailty. This limitation increases the risk of undertriage and delays in appropriate care for older adults [6–8]. Research has shown that standard triage systems are not tailored to the needs of frail older patients and often have reduced predictive value for short-term mortality and morbidity in this group [6, 9–13].

Clinical assessment based on chief complaint, vital sign and triage are central in the ED yet the association between vital signs and outcomes can vary significantly between different age groups. Research examining the relationship between systolic blood pressure (SBP) and trauma shows that undertriage is a concern when using common triage systems and cut-off limits for older adults [14, 15]. Similar findings regarding SBP are shown for elderly patients presenting to the ED with a suspected infection [16]. Other studies have shown that level of consciousness (LOC) and peripheral oxygenation (SpO₂) appears to have the strongest correlation to mortality early after triage [17]. Age is rarely taken into consideration in triage even though research has shown a correlation between older patients and mortality [17, 18].

Different studies have demonstrated the significance of age and vital signs in triage decision-making in the ED and the need for age-adjusted criteria’s. There is a need to understand how vital signs influence outcomes in older adults.

The aim of this study was to explore whether different vital signs used in ED triage were associated with 7-day mortality in elderly patients visiting the ED.

## Methods

### Setting and design

This retrospective observational study included patients *≥* 65 years of age visiting one of the two ED in Region Halland (RH) between 1st of January 2018 and 31st of December 2018. RH is located on the southwest coast of Sweden with a population of approximately 330,000, where 23% were older adults aged ≥ 65 years. The region had 468 hospital beds during the study period. Patients who arrive at the ED routinely present their chief complaint and are triaged according to RETTS, which is commonly used in Sweden [19]. In RETTS, triage levels are color-coded to indicate clinical urgency. Red represents life-threatening conditions requiring immediate attention, orange denotes very urgent cases, yellow indicates urgent but stable conditions, green is used for less urgent issues, and blue applies to non-urgent cases. The classification is based on vital signs and presenting symptoms to guide prioritization of care [20].

### Patients and selection

During the study period, all patients aged over 65 years who visited either of the two EDs in RH, Sweden were included if they had at least one recorded vital sign—SBP, heart rate (HR), SpO₂, respiratory rate (RR), body temperature, or LOC. Patients with no documented vital signs were excluded from the study.

### Data collection

Data was generated from the Regional Healthcare Information Platform (RHIP) [21]. RHIP links pseudo-anonymized data regarding healthcare encounters and healthcare utilization in the region using routinely collected data during standard care. Variables extracted were age, gender, comorbidities before visits to the ED. The included individuals were grouped by age into three categories: 65–74 years, 75–84 years, and 85 years or older. Comorbidities were calculated using Charlson Comorbidity Index (CCI). Comorbidities was based on all primary and secondary diagnoses across visits to all caregivers and divided into mild (CCI score 1–2), moderate (CCI score 3–4) and severe (CCI score ≥ 5) [22]. All diagnoses were registered according to the International Classification of Disease-10 (ICD-10). In addition, chief complaint, mode and time of arrival along with triage level and vital signs were collected for each patient. Chief-complaint for ED-visits were collected from electronic medical records. Fatigue, confusion, non-specific complaints, generalized weakness and risk of falling was defined as non-specific complaint (NSC) [23]. Outcomes following ED arrival were obtained from RHIP, including triage level, ED length of stay (LOS), hospital admission rate, total bed-days, 72-hour revisit rate, and 7-day mortality. Data on 7-day mortality were collected for all patients, regardless of whether they were admitted to the hospital or discharged from the ED. Number of bed-days was calculated on patients in the population that was admitted to the hospital after visiting the ED. A 72-hour revisit was defined as a return visit to one of the two ED in RH within 72 h of the initial visit, regardless of the presenting complaint.

Vital signs were registered in triage on arrival at the ED according to RETTS, only one set of vital signs were collected for each patient. Vital signs recorded were SBP (mmHg), HR, (beats/min), SpO2 (%), RR (/min), temperature (°C) and LOC. In the ED setting for this study, LOC was assessed using the Reaction Level Scale (RLS), which is routinely used in many EDs across Scandinavia. RLS ranges from 1 (fully alert) to 8 (no reaction), with higher scores indicating more severe impairment [24]. To improve international comparability, RLS scores were converted to Glasgow Coma Scale (GCS) categories based on previously published evidence [25, 26]. The following approximation was applied: RLS 1 → GCS 13–15 (mild or no impairment), RLS 3 → GCS 9–12 (moderate impairment), and RLS 4–8 → GCS 3–8 (severe impairment). This stratification enabled classification of consciousness levels using internationally recognized GCS thresholds while preserving clinically meaningful distinctions relevant to emergency care.

### Outcome measures

The primary outcome measure was 7-days mortality after visiting the ED. Secondary outcome measures were ED LOS, admission and in-hospital LOS.

### Statistical analysis

Descriptive statistics were used to summarize patient characteristics and vital signs. Continuous variables were presented as means with standard deviations (SD) and analysed using one-way ANOVA, while categorical variables were reported as frequencies and percentages.

Normality of continuous variables (SBP, HR, SpO₂, RR, temperature, and LOC) was assessed through visual inspection of histograms and by evaluating skewness and kurtosis. These methods provided insight into data symmetry, variability, and the presence of outliers. Although some variables deviated from a normal distribution, the large sample size justified the use of parametric methods. According to the Central Limit Theorem, the sampling distribution of the mean approximates normality as sample size increases. Therefore, t-tests and ANOVA were considered appropriate and robust for analysing group differences in this dataset [27].

To explore the predictive performance of individual vital signs for 7-day mortality, Receiver Operating Characteristic (ROC) curve analysis was performed. The Area Under the Curve (AUC) quantified each variable’s ability to discriminate between survivors and non-survivors, with higher values indicating greater predictive accuracy [28].

To enhance interpretability and reflect clinical practice, vital signs were categorized based on clinically established thresholds and cut-offs used in previous studies [11]. Categories were defined as follows: SBP (*≤* 80, 81–100, 101–120, 121–140, 140–160, *≥* 160 mmHg), HR (*≤* 50, 51–75, 76–100, 101–125, > 125 bpm), SpO₂ (*≤* 80, 81–85, 86–90, 91–95, 96–100%), RR (*≤* 9, 10–19, 20–29, *≥* 30 breaths/min), temperature (*≤* 30 °C, 31–34 °C, 35–37 °C, 38–39 °C, *≥* 40 °C), and LOC (GCS 3–8, 9–12, 13–15). A reference category was defined for each vital sign. Reference categories for vital sign variables were chosen to support a logical, stepwise progression across risk levels in the regression analysis, rather than using clinically “normal” ranges, in order to better reflect the relationship between physiological extremes and mortality risk [29]. Missing values were categorized as “missing data”.

Binary logistic regression was used to assess associations between categorized vital signs and 7-day mortality. Univariable models estimated crude effects, while multivariable models were adjusted for age, sex and comorbidities. Results were reported as odds ratios (OR) and adjusted odds ratios (AOR) with 95% confidence intervals (CI). To ensure model validity, multicollinearity among independent variables was assessed using Variance Inflation Factor (VIF) and Tolerance statistics. VIF values < 5 and Tolerance values > 0.1 were considered acceptable. A p-value of < 0.05 was considered statistically significant. The analyses were executed with IBM SPSS Statistics 27, Armonk, New York, USA. There were no missing values besides from vital signs which are presented in Table 1.

**Table 1:** Baseline characteristics of the study population upon arrival to the ED

	Total	65–74 years	75–84 years	*≥* 85 years	*p*-value
**Total**,** n (%)**	25 450 (100)	9669 (38)	9747 (38)	6034 (24)	
**Demographics**,** n (%)**					
Sex, female	12 812 (50)	4603 (48)	4767 (49)	3442 (57)	< 0.001
**Charlson Comorbidity Index**					< 0.001
Mild	2119 (8)	2119 (22)	0 (0)	0 (0)	
Moderate	10 814 (43)	5245 (54)	4180 (43)	1389 (23)	
Severe	12 517 (49)	2305 (24)	5567 (57)	4645 (77)	
**Arrival by ambulance**,** n (%)**	10 391 (41)	2613 (27)	4049 (41)	3729 (62)	< 0.001
**Arrival time of day**,** n (%)**					0.004
08.00–16.00	14 349 (56)	5360 (55)	5610 (58)	3379 (56)	
16.00–20.00	8173 (32)	3152 (33)	3019 (31)	2002 (33)	
20.00–08.00	2928 (12)	1157 (12)	1118 (11)	653 (11)	
**Triage level**,** n (%)**					< 0.001
Blue	293 (1)	144 (2)	104 (1)	45 (1)	
Green	2467 (10)	1142 (12)	890 (9)	435 (7)	
Yellow	11 621 (46)	4789 (50)	4399 (45)	2433 (40)	
Orange	10 278 (40)	3316 (34)	4060 (42)	2902 (48)	
Red	701 (3)	228 (2)	258 (3)	215 (4)	
Missing data	90 (0)	50 (0)	36 (0)	4 (0)	
**Vital signs**,** n (%)**					
*SBP (mmHg)*					< 0.001
>160	5453 (21)	2099 (22)	2065 (21)	1289 (21)	
141–160	6869 (27)	2768 (29)	2651 (27)	1450 (24)	
121–140	5213 (21)	2001 (21)	1966 (20)	1246 (21)	
101–120	5110 (20)	1725 (18)	1997 (21)	1388 (23)	
81–100	1016 (4)	305 (3)	411 (4)	300 (5)	
*≤* 80	114 (0)	32 (0)	50 (1)	32 (1)	
Missing data	1675 (7)	739 (7)	607 (6)	329 (5)	
*Heart rate (beats/min)*					< 0.001
*≤* 50	391 (2)	114 (1)	159 (2)	118 (2)	
51–75	9399 (37)	3376 (35)	3665 (38)	2358 (39)	
76–100	10 637 (42)	4119 (43)	4005 (41)	2513 (42)	
101–125	2920 (11)	1173 (12)	1120 (11)	627 (10)	
>125	851 (3)	308 (3)	346 (3)	197 (3)	
Missing data	1252 (5)	579 (6)	452 (5)	221 (4)	
*SpO*₂ *(%)*					< 0.001
96–100	17 974 (70)	7231 (75)	6823 (70)	3920 (65)	
91–95	5209 (20)	1568 (16)	2057 (21)	1584 (26)	
86–90	729 (3)	211 (2)	303 (3)	215 (4)	
81–85	157 (1)	47 (1)	75 (1)	35 (0)	
*≤* 80	143 (1)	38 (0)	58 (1)	47 (1)	
Missing data	1238 (5)	574 (6)	431 (4)	233 (4)	
*Respiratory rate (/min)*					< 0.001
*≤* 9	70 (0)	21 (0)	31 (0)	18 (0)	
10–19	15 091 (59)	6133 (63)	5753 (59)	3205 (53)	
20–29	7654 (30)	2492 (26)	3011 (31)	2151 (36)	
*≥* 30	678 (3)	189 (2)	239 (3)	250 (4)	
Missing data	1957 (8)	834 (9)	713 (7)	410 (7)	
*Temperature (°C)*					0.02
*≤* 30	7 (0)	5 (0)	1 (0)	1 (0)	
31–34	61 (0)	19 (0)	18 (0)	24 (0)	
35–37	20 423 (80)	7823 (81)	7875 (81)	4725 (78)	
38–39	1393 (6)	572 (6)	530 (5)	291 (5)	
*≥*40	21 (0)	7 (0)	10 (0)	4 (0)	
Missing data	3545 (14)	1243 (13)	1313 (14)	989 (16)	
*LOC (GCS)*					< 0.001
13–15	17 782 (70)	6954 (72)	6824 (70)	4004 (66)	
9–12	101 (0)	24 (0)	42 (0)	35 (1)	
3–18	71 (0)	26 (0)	20 (0)	25 (0)	
Missing data	7496 (30)	2665 (28)	2861 (30)	1970 (33)	

Note: n = number, ED = emergency department, SpO₂ = oxygen saturation, SBP = systolic blood pressure, LOC = levelof consciousness, GCS = Glasgow Coma Scale

## Results

### Study population and baseline characteristics

**Fig. 1:**
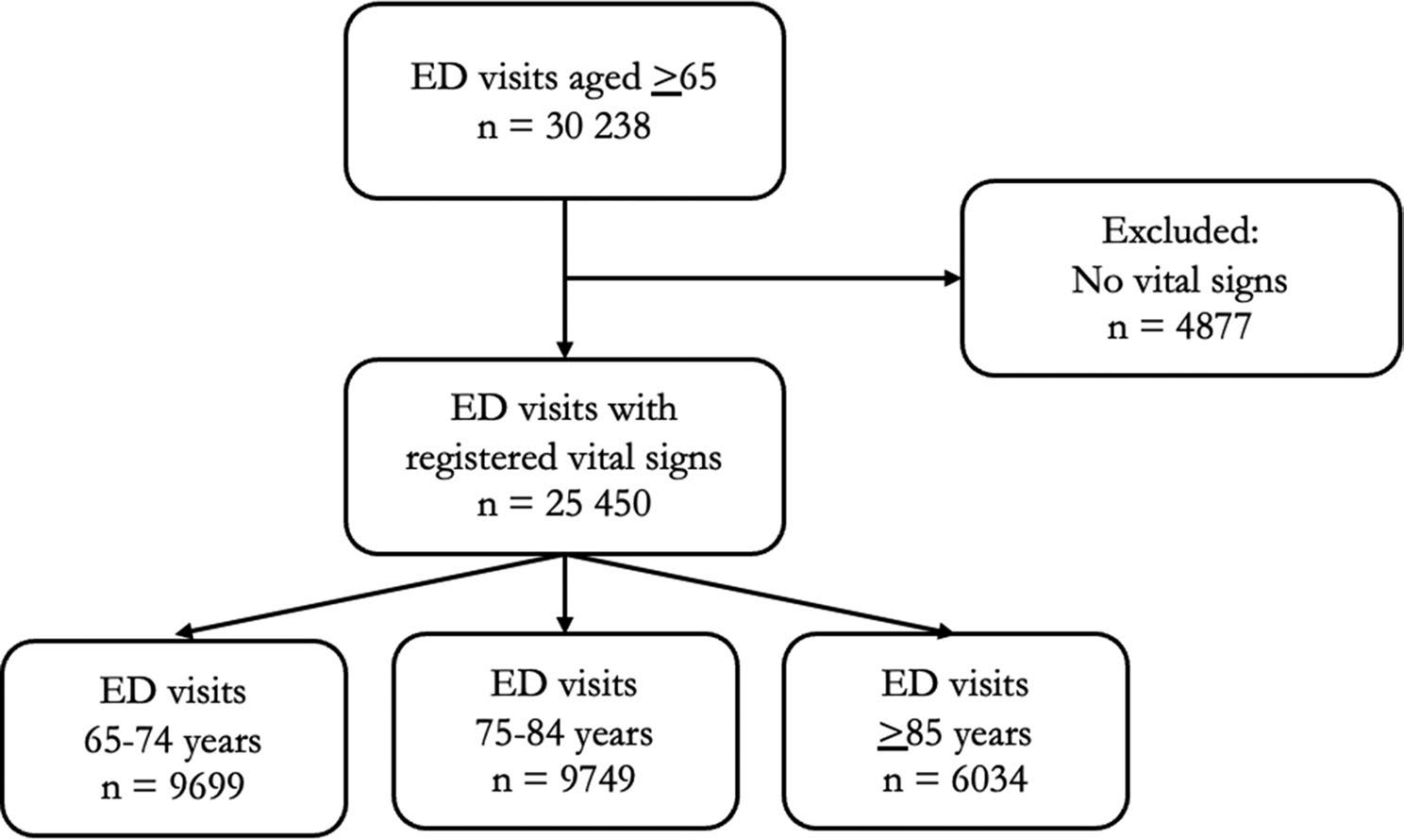
Patient inclusion flow-chart. Note: ED = Emergency Department, n = numbers

During the study period, a total of 30 327 patients aged over 65 years visited one of the two EDs in RH, Sweden. Of these, 25 450 patients with at least one recorded vital sign—SBP, HR, SpO₂, RR, body temperature, or LOC—were included in the study. Patients without any recorded vital signs were excluded, as illustrated in Fig. 1.

The mean age of the study population was 78 years (SD 8.1), with 12 812 individuals (50%) identified as female. There were 23 331 (92%) individuals classified as having moderate to severe comorbidity according to CCI. Table 1 presents the baseline characteristics of the study population on arrival to the ED. Variables include sex, comorbidities (based on CCI), mode of arrival (ambulance vs. other), time of arrival (categorized as day, evening, or night), triage level according to RETTS, and initial vital signs (SBP, HR, SpO2, RR, temperature, and LOC).

### ED utilization and patient outcomes

A total of 12 807 patients (50%) spent more than four hours in the ED, and 13 937 (55%) were admitted to the hospital. Admission rates increased with age, reaching 70% in patients over 85 years. One-way ANOVA revealed a statistically significant difference in in-hospital LOS across age groups. The overall mean LOS was 2.6 days (SD 4.3), increasing from 1.8 days in patients aged 65–74 to 3.7 days in those over 85. The 72-hour ED revisit rate was low (4%), likely reflecting the high admission rate. A total of 483 patients (2%) died within 7 days of their ED visit. Among those who died, 437 (90%) arrived by ambulance, 151 (31%) spent more than four hours in the ED, and 433 (90%) were admitted to hospital. Most of these patients, 339 (70%), had been triaged as yellow or orange on arrival.

Among patients who died within 7 days of their ED visit, the most common chief complaint was dyspnea, accounting for 31% of cases. This was followed by abdominal pain (12%), NSC (10%), fever or suspected infection (8%), trauma (6%), stroke (6%), chest pain (5%), cardiac arrest (5%), syncope (4%), and extremity problems (1%).

### Association between vital signs and 7-Day mortality

The mean vital signs for the study population were: SBP 142 mmHg, HR 82 beats/min, SpO₂ 96%, RR 18 breaths/min, and body temperature 36.8 °C. LOC was not recorded in 7,496 cases (29%), while 17 782 patients were assessed as GCS 13–15 (mild or no impairment). The distribution of vital signs across age groups is presented in Table 1.

Predictive performance of individual vital signs for 7-day mortality based on ROC curve analysis are presented in Table 2. SBP demonstrated the highest discriminatory ability (AUC 0.70), followed by RR (AUC 0.69), and SpO₂ (AUC 0.68). While these values indicate moderate predictive performance, both sensitivity (56–58%) and specificity (74–79%) remained limited. HR and LOC showed lower discriminative performance (both AUC 0.64). GCS > 9 had a high specificity (99%) but low sensitivity (17%). Temperature showed no meaningful discriminative value (AUC 0.49). Across all variables, Youden’s Index values were modest (ranging from 0.13 to 0.35), indicating that no single vital sign provided sufficient discriminatory power for accurate risk stratification.

**Table 2:** Predictive performance of individual vital signs for 7-day mortality based on ROC curve analysis

Variable	AUC	95% C.I	*p*-value	Cut-off value	Sensitivity	Specificity	Youden’s Index
SBP	0.70	0.67–0.72	< 0.001	125	58%	74%	0.33
Respiratory rate	0.69	0.66–0.72	< 0.001	20	56%	79%	0.35
SpO₂	0.68	0.66–0.71	< 0.001	95	56%	75%	0.31
Heart rate	0.64	0.61–0.67	< 0.001	92	51%	73%	0.24
GCS	0.64	0.55–0.62	< 0.001	> 9	17%	99%	0.16
Temperature	0.49	0.46–0.52	0.02	36,5	27%	86%	0.13

Note: SBP = systolic blood pressure, SpO₂ = oxygen saturation, LOC = level of consciousness, AUC = Area under curve

Multivariable linear regression analysis showed that lower SBP (β = − 0.064), lower SpO2 (β = − 0.084), higher HR (β = 0.042), higher RR (β = 0.061), and lower body temperature (β = − 0.032) were all independently associated with increased 7-day mortality (all *p* < 0.001). Impaired LOC showed the strongest association with mortality (β = 0.183, *p* < 0.001). Variance Inflation Factor values were all below 1.2, indicating no statistically significant multicollinearity among predictors, confirming the robustness of the model.

As shown in Table 3, multivariable logistic regression analysis identified increasing age as a consistent predictor of 7-day mortality (AOR 1.08, 95% CI 1.06–1.10, *p* < 0.001), while sex was not statistically significantly associated with mortality (AOR 1.02, 95% CI 0.77–1.35, *p* = 0.91). Comorbidity burden, as measured by CCI, was associated with higher mortality risk, though confidence intervals for moderate and severe CCI levels were wide and did not reach statistical significance.

**Table 3:** The association between vital signs and 7-days mortality. Unadjusted and adjusted odds ratio

	Univariable analysis			Multivariable analysis		
	OR	95% C.I	*p*-value	AOR	95% C.I	*p*-value
**Age**	1.09	1.08–1.10	< 0.001	1.08	1.06–1.10	< 0.001
**Sex**,** female**	0.96	0.80–1.15	0.66	1.03	0.78–1.37	0.84
**Comorbidity (CCI)**						
Mild	Ref.		< 0.001	Ref.		0.06
Moderate	3.83	1.56–9.41		4.17	0.56–31.03	
Severe	13.27	5.49–32.11		5.78	0.77–43.10	
**SBP (mmHg)**						
*≥* 160	Ref.		< 0.001	Ref.		< 0.001
141–160	0.94	0.64–1.37		0.80	0.48–1.34	
121–140	1.62	1.13–2.32		1.27	0.78–2.06	
101–120	3.51	2.55–4.84		1.98	1.28–3.06	
81–100	8.36	5.80–12 − 07		3.73	2.22–6.28	
*≤* 80	31.93	19.00–53.63		7.79	3.27–18.54	
**Heart rate (beats/min)**						
*≤* 50	Ref.		< 0.001	Ref.		< 0.001
51–75	0.54	0.26–1.12		2.26	0.47–10.83	
76–100	0.87	0.42–1.77		3.17	0.67–15.08	
101–125	1.96	0.95–4.05		6.36	1.32–30.50	
>125	2.61	1.22–5.60		6.37	1.26–32.16	
**SpO2 (%)**						
96–100	Ref.		< 0.001	Ref.		< 0.001
91–95	2.54	2.05–3.15		1.36	0.98–1.89	
86–90	8.47	6.33–11.33		2.93	1.83–4.70	
81–85	9.29	5.36–16.11		2.23	0.93–5.33	
*≤* 80	22.38	14.55–34.42		3.64	1.75–7.58	
**Respiratory rate (/min)**						
*≤* 9	Ref.		< 0.001	Ref.		< 0.001
10–19	0.05	0.02–0.09		0.27	0.05–1.51	
20–29	0.14	0.07–0.26		0.51	0.09–2.84	
*≥* 30	0.66	0.34–1.29		0.88	0.15–5.05	
**Temperature (°C)**						
*≤* 30	Ref.		< 0.001	Ref.		< 0.001
31–34	0.61	0.11–3.55		0.26	0.01–7.02	
35–37	0.04	0.01–0.21		0.03	0.00–0.81	
38–39	0.07	0.01–0.39		0.02	0.00–0.62	
*≥*40	0.12	0.01–1.67		0.00		
**Level of consciousness (GCS)**						
13–15	Ref.		< 0.001	Ref.		< 0.001
9–12	22.69	14.10–36.50		9.30	4.97–17.39	
3–8	44.66	27.21–73.32		18.36	7.95–42.40	

Note: OR = Odds Ratio, AOR = Adjusted Odds Ratio, CCI = Charlson Comorbidity Index, SBP = Systolic blood pressure, Sp02 = Peripheral Oxygen Saturation, RLS = Reaction Level Scale

Compared to SBP > 160 mmHg, SBP values of 101–120 mmHg (AOR 1.98, 95% CI 1.28–3.06), 81–100 mmHg (AOR 3.73, 95% CI 2.22–6.28), and ≤ 80 mmHg (AOR 7.79, 95% CI 3.27–18.54) were statistically significantly associated with increased odds of death.

Heart rate *≥* 101 bpm and SpO₂ ≤90% were also independently associated with increased odds of death. While RR and temperature contributed to the overall model, no individual category reached statistical significance. Reduced LOC (GCS) remained a robust predictor of 7-day mortality: patients with GCS 9–12 (AOR 9.30, 95% CI 4.97–17.39) and GCS 3–8 (AOR 18.36, 95% CI 7.95–42.41) had markedly elevated odds of death compared to those with CGS 13–15.

## Discussion

### Principal findings

This study examined the association between initial vital signs and 7-day mortality in a large cohort of older adults presenting to two EDs in RH, Sweden. The findings demonstrate that several vital signs, when categorized, were independently associated with short-term mortality. However, their predictive performance was modest when evaluated in isolation, reinforcing the need to interpret vital signs in the broader context of age and comorbidities.

### Interpretation in context

Consistent with prior research, lower SBP, elevated HR, reduced SpO₂, and impaired LOC were all independently associated with increased risk of 7-day mortality [30–32]. SBP showed a strong inverse relationship with mortality risk: patients with SBP < 120 mmHg had a progressively higher odds of death, with the highest risk observed in those with SBP *≤* 80 mmHg. This aligns with existing literature suggesting that even moderate hypotension older adults may reflect inadequate perfusion or serious underlying pathology [33, 34].

Tachycardia (HR > 100 bpm) and hypoxemia (SpO₂ *≤*90%) were also strongly associated with mortality, supporting their value as early warning indicators in emergency triage. Impaired LOC, assessed using GCS (converted from RLS), emerged as the strongest individual predictor of mortality. Both moderate (GCS 9–12) and severe impairment (GCS 3–8) were associated with markedly elevated odds of death, underscoring the critical role of mental status evaluation in geriatric emergency care [35, 36].

RR and temperature were statistically significant in the overall multivariable model but did not show a statistically significant association at the category level. This pattern may reflect interactions with other physiological variables or the complexity of altered vital signs in older adults.

### Predictive performance and clinical implications

The ROC curve analysis confirmed the limited discriminatory power of individual vital signs. SBP had the highest AUC (0.70), RR (0.69), and SpO₂ (0.68). Despite these relatively higher values, sensitivity and specificity remained modest, and Youden’s Index values were uniformly low (ranging from 0.13 to 0.35), indicating limited standalone predictive value. LOC and HR had lower AUC values (both 0.64), and temperature showed no meaningful discriminative ability (AUC 0.49).

These findings support the use of categorized vital signs in risk modelling, which better reflects real-world clinical practice where decisions often rely on defined thresholds rather than continuous values. The modest predictive power of individual parameters emphasizes the importance of incorporating multiple factors into triage decisions.

Our findings reinforce the need for age-adapted and multifactorial risk stratification tools in emergency care. While traditional vital signs remain essential, their interpretation in older patients should always be contextualized alongside age, comorbidities and LOC. Future triage systems may benefit from incorporating composite scoring models or geriatric-specific indicators that better reflect the complexity and clinical heterogeneity of this vulnerable group.

## Limitations

This study benefits from a large sample size from two unselected EDs, enhancing the reliability and generalizability of the findings. The inclusion of real-world data from a single region minimizes selection bias, as no data was collected from other hospitals or regions. Since all patients visiting the ED in the region were eligible for inclusion, the study group is considered representative of the general population in Sweden and likely Scandinavia.

Several limitations should be noted. First, this study used retrospective registry data, which may be subject to documentation bias or missing data, especially for LOC, which was not recorded in nearly one-third of patients. Second, while the study focused on associations with 7-day mortality, it did not account for cause-specific mortality or long-term outcomes. Third, we did not include functional status, frailty indices, or medication use, which are known to influence outcomes in older populations. Additionally, although the RHIP system integrates data across care levels within RH, re-visits or deaths occurring outside the region may have been missed, although likely few in number. Since frailty is often not routinely documented in most ED settings in Sweden, it could not be included in the analysis. To partially address this limitation, comorbidities were used as a proxy for underlying health conditions and frailty.

Furthermore, the data were collected in 2018, prior to the COVID-19 pandemic. Since then, both patient profiles and emergency care practices may have changed, including increased use of telemedicine, modifications to triage protocols, and shifts in healthcare-seeking behaviour. These factors may influence the generalizability of the findings to current clinical settings.

It should be emphasized that the results in this study are associations, and it is not possible to draw any conclusions regarding causality.

## Conclusion

In this large cohort of older ED patients, several initial vital signs, particularly low SBP, elevated HR, reduced SpO₂, and impaired LOC, were independently associated with 7-day mortality. No single parameter demonstrated sufficient discriminative performance to serve as a standalone predictor of short-term mortality. LOC, while a strong prognostic predictor, was frequently missing, highlighting challenges in routine assessment. These findings underscore the need for multifactorial risk stratification in geriatric emergency care. As key predictors like frailty and functional status were not available, future studies should focus on developing integrated triage models that incorporate age-related physiological changes, comorbidity burden and frailty to improve early identification of high-risk older adults and support clinical decision-making in emergency care.

## Data Availability

The datasets generated and/or analysed during the current study are not publicly available due to the data being retrieved from patient’s hospital records which is included in the Swedish Health Care act which applies to Swedish secrecy act according to Swedish legislation. The data will be shared on reasonable request to the corresponding author. Clinical trial number: not applicable.

## Abbreviations

AOR: Adjusted Odd’s Ratio
CCI: Charlson Comorbidity Index
ED: Emergency Department
GCS: Glasgow Coma Scale
HR: Heart rate
ICD-10: International Classification of Disease-10
LOC: Level of consciousness
LOS: Length of stay
NSC: Non-specific complaint
OR: Odd’s Ratio
Older adults: 65 years of age and above
RLS: Reaction Level Scale
RR: Respiratory rate
RETTS-A: Rapid Emergency Triage and Treatment System-Adult
RH: Region Halland
RHIP: Region Healthcare Information Platform
RLS: Reaction Level Scale
SBP: Systolic Blood Pressure
SD: Standard Deviation
SpO₂: Peripheral Oxygen Saturation
